# A data linkage strategy for producing census and population statistics from administrative data

**DOI:** 10.23889/ijpds.v1i1.378

**Published:** 2017-04-19

**Authors:** Pete Jones

**Affiliations:** 1 Office for National Statistics

## Objectives

Following the recommendation of the National Statistician in 2014, it is intended that the 2021 Census of England and Wales will make far greater use of administrative data. The combined use of administrative and census data has the potential to enhance the quality and detail of outputs that can be produced in 2021. Furthermore, the government's aspiration is that future censuses will be conducted with other sources of data. One of the major objectives of the next census is therefore to develop and test methods for producing a future alternative that relies primarily on administrative data and surveys. 

## Approach

In order to meet the objectives of the 2021 Census, a data linkage strategy is needed to support the statistical system for producing population statistics. Given the diverse uses of linked data in census statistical processing, each matching exercise will have different requirements in terms of scale, methodology and quality. This paper outlines a flexible methodological strategy that has been developed to meet those requirements, with examples of research that has been undertaken to date.

## Results

Research findings from a range of linkage exercises are presented with discussion around the methods used, the scale of the matching exercise and associated measures of quality. Examples include:

- Linking multiple administrative datasets to produce a `Statistical Population Dataset'
- Linking to adjust for coverage errors using capture-recapture methods
- Generating multivariate tabulations from linked administrative and survey data
- Using linked administrative data to improve item imputation for missing values
- Linking of address records to assign Unique Property Reference Numbers
- Using administrative data to enhance the 2021 Census Address Register

## Conclusion

Central to the strategy is the need to develop a business model that can deliver linkage outputs to the required quality while still preserving the privacy of individuals' data. We conclude that various procedural and technical options for preserving privacy can be incorporated within the framework of this strategy, including pseudonymisation, de-identification, trusted third party models and record indexing. The strategy developed will enable datasets to be linked to the required specifications. In addition, de-identified datasets can be held separately and integrated efficiently when required in the production of statistical outputs.

The development of this strategy will continue in the run up to the 2021 Census, with the aim of incorporating its use in wider statistical output production, including population, business statistics and social surveys.

